# Coronary Computed Tomography Angiography with Deep Learning Image Reconstruction: A Preliminary Study to Evaluate Radiation Exposure Reduction

**DOI:** 10.3390/tomography9030083

**Published:** 2023-05-16

**Authors:** Rossana Bona, Piergiorgio Marini, Davide Turilli, Salvatore Masala, Mariano Scaglione

**Affiliations:** 1 Medical Physics Unit, Azienda Ospedaliero-Universitaria (AOU), 07100 Sassari, Italy; 2 Department of Medicine, Surgery and Pharmacy, University of Sassari, 07100 Sassari, Italy

**Keywords:** ionizing radiation exposure, coronary vessels, low-dose computed tomography, deep learning algorithm, exam optimization

## Abstract

Coronary computed tomography angiography (CCTA) is a medical imaging technique that produces detailed images of the coronary arteries. Our work focuses on the optimization of the prospectively ECG-triggered scan technique, which delivers the radiation efficiently only during a fraction of the R–R interval, matching the aim of reducing radiation dose in this increasingly used radiological examination. In this work, we analyzed how the median DLP (Dose-Length Product) values for CCTA of our Center decreased significantly in recent times mainly due to a notable change in the technology used. We passed from a median DLP value of 1158 mGy·cm to 221 mGy·cm for the whole exam and from a value of 1140 mGy·cm to 204 mGy·cm if considering CCTA scanning only. The result was obtained through the association of important factors during the dose imaging optimization: technological improvement, acquisition technique, and image reconstruction algorithm intervention. The combination of these three factors allows us to perform a faster and more accurate prospective CCTA with a lower radiation dose. Our future aim is to tune the image quality through a detectability-based study, combining algorithm strength with automatic dose settings.

## 1. Introduction

Cardiac computed tomography (CCT) is a relatively non-invasive (because of the use of X-ray and venous iodine contrast) and low-cost (with respect to coronary angiography) diagnostic tool that was introduced in the 1990s to identify and quantify coronary artery stenosis. Subsequently, the use of CCTA has become appropriate in different clinical scenarios and is now recommended as a first-line diagnostic test in many situations (for example, primary prevention of coronary disease in asymptomatic patients; evaluation of patients with chronic coronary syndrome, acute chest pain, or previous coronary revascularization; and assessment of cardiomyopathies, structural heart disease, and congenital heart disease). Moreover, it has grown into a global imaging modality as it offers an anatomical and functional evaluation of the structure of the heart, which can be used to guide the clinical care of selected patients.

A tumultuous technological development has reduced acquisition examination time, which is critical for imaging a moving structure, and has provided improved image quality while reducing radiation exposure among patients.

The state of the art of high-definition CCTA allows a complete assessment of coronary artery disease as it provides excellent image quality of blood vessels, including those in the periphery. 

In addition to the high accuracy in detecting significant coronary stenosis, it allows the assessment of morphology and characterization of vascular plaque composition. Therefore, CCTA has become a valid alternative to the more invasive and expensive conventional coronary angiography in the study of suspected coronary artery disease [1].

Deep Learning Image Reconstruction (DLIR) algorithms contribute to this result. 

In our hospital, the purchase of a new AI-based technology was driven by an obsolete in-house technique and by the need to use algorithms available on the market that have really changed the perspective of radiological diagnosis. DLIR is developed as a subset of artificial intelligence (AI) algorithms, and it is the crucial factor that can contribute to improving image quality and reducing radiation dose at the same time, above all because improvements in scanner mechanics must wait for the development and diffusion of photon counting techniques. An international dose survey proves that radiation exposure associated with cardiovascular CT has been tremendously reduced by 78% over the last decade [2]. This is the gold standard to obtain diagnostic targets when aiming to reduce stochastic risk.

The aim of this study is to evaluate image quality improvement and dose reduction after our hospital’s technological update.

We firstly compared the most relevant acquisition parameters and dosimetric quantities in prospective CCTA obtained from a previous scanner (Philips iCT—128 slices) equipped with the FBP algorithm and the recently acquired one (GE Revolution—256 slices), which reconstructs raw data using both the model-based Adaptive Statistical Iterative Reconstruction-Veo (ASIR-V) and the Deep Learning Image Reconstruction (DLIR) algorithm, TrueFidelity™.

We then looked at the Diagnostic Reference Levels (DRLs) found in the literature, as they represent the primary tool to optimize radiological practice. Our Dose team, comprised both radiologists and medical physicists, also performed a qualitative assessment of the images and then a trough phantom analysis.

Moreover, the acquisition of a dose monitoring system, Radimetrics Enterprise Platform™ (version 3.4.2B), allowed us to obtain an accurate estimation of the effective doses and the equivalent doses to the heart via the use of a Monte Carlo simulation software. We validated the results against the previous evaluation method using statistical analyses according to the *p*-value.

## 2. Materials and Methods

### 2.1. Study Population

We carried out a retrospective analysis on two groups of patients scanned at the Azienda Ospedaliero-Universitaria (AOU) Hospital of Sassari. For both groups, the inclusion criterion was patients undergoing diagnostic prospective CCTA for coronary artery disease. After the anonymization of the examinations, we collected the remaining personal data and dosimetric quantities of all irradiation events.

First, we extracted data from 20 patients (Group A) examined from September 2021 to September 2022 with the iCT Philips scanner.

Then, we collected data from 20 patients (Group B) examined from October 2022 to January 2023 with the GE Revolution CT. 

Another criterion for the inclusion of patients was the parameter BPM (beats per minute), with the condition that it did not exceed 65. This criterion was closely linked to the need for an absence of artefacts due to organ motion during image acquisition, and it was essential to obtain good image quality, as well as to determine diagnostic significance. For this purpose, if necessary, beta-blockers were administered.

For Group B, this criterion regarding BPM was less critical as the advanced scanner compensates for more frequent and irregular beats.

As a further aid to ensure the quality of the examination, sublingual isosorbide dinitrate was administered. It is a blood vessel dilator that improves the induced blood flow in the coronary bed, and, consequently, image quality in terms of motion artifact reduction.

The exclusion criterion for both groups was patients with images of poor quality that could not be accurately reported.

### 2.2. Image Acquisition 

The patients included in this study were all asked to be in the supine position with their arms raised and their hands resting on the head.

ECG gating was used to acquire images at the diastolic phase when the contrast agent had fully perfused in the coronary arteries. Cardiac prospectively ECG-gated scan mode involves image acquisition in which a trigger is obtained from the ECG signal to start the scan during a cardiac phase with minimal movement [3].

Initially, the mean duration of an RR interval (the time elapsed between two successive R-waves of the Quality Rating System signal on the electrocardiogram) was calculated as the average of several cardiac cycles.

A sequential scan was then initiated at a predefined time in each subsequent cardiac cycle. This trigger point was placed, in our system, within the diastolic phase of the cardiac cycle defined by the examiner as a percentage of the RR interval (relative trigger).

After the pre-monitoring phase (Smart Prep), the scanning region in the CCTA covered the entire coronary circulation and the heart. 

As shown in Table 1, Group A patients were scanned using a helical acquisition technique with the following parameters: 64 × 0.625 mm detector collimation, a tube voltage variable between 120 and 140 kV, a pitch factor of 0.18, a medium tube time rotation of 1.7 s, and an almost always fixed tube current with very high mAs values. 

The technological limits made it necessary to deliver the radiation during a large R–R interval and with a fixed reconstruction in the best cardiac phase as chosen by the system. It was possible to obtain sufficient image quality only for patients with heart rates ≤ 65 bpm.

Group B patients were scanned using the axial acquisition technique with the following parameters: 256 × 0.625 mm collimation, a tube voltage variable between 100 and 120 kV, a tube rotation time of 0.28 s, and a mean value of 155 mAs for the automatic modulated tube current. 

The system’s collimation coverage of 160 mm on a fixed bed position was sufficient to perform the entire exam with a single rotation of the tube. 

The machine-selected protocol (CCTA protocol with no auto-gating) makes use of the ECG-gated mode with image acquisition between 40 and 80% of the cardiac cycle. Reconstruction is set automatically at 75% cardiac phase, leaving the user with the possibility of reconstructing any phase in a retrospective way [4].

The user can even specify the cardiac cycle phases to be acquired and the relative tube current level for each part of this acquisition window. The system responds to the patient’s heart rate and modulates the tube current to capture the heart rates’ phases as requested by the user, while reducing the dose administered to the patient. In addition, it automatically indicates the approximate range of phases available for image reconstruction at 100% mA value [4].

### 2.3. Image Reconstruction

Despite the mechanical and constructive improvements of CT scanners, i.e., the increase in the rotation speed of their tube/detector system, the CT reconstruction algorithm used is a crucial factor affecting radiological diagnosis. 

The appearance of the images reconstructed using the FBP is certainly highly appreciated by radiologists; however, this comes at the cost of a high intake of radiation doses. In fact, the input sinogram of the FBP is accurate when radiation dose is high, but in low-dose settings, it is challenged by the higher image noises and artifacts.

Various attempts to reduce radiation dose with our old scanner led to excessive noise growth, thereby heavily affecting the diagnosis. 

The introduction of iterative algorithms (IR) makes it possible to reduce noises, increase low-contrast detectability, and lower radiation doses, within certain limits. 

In short, IR integrates matrix algebra to convert the measured value of each pixel to a new estimated one. This pixel value is then evaluated and compared with the ideal value that is predicted using noise modeling.

This process is iterated until converging to the final estimated ideal pixel values. As is well known, an excessive intervention of these algorithms considerably modifies the appearance of the images, risking a drop in the spatial resolution for low-contrast structures or a “blotchy” noise texture [5].

In our scanner, the GE iterative algorithm ASIR-V contains advanced noise, object, and physics modeling. The model assumes a three-dimensional volume of each voxel element and takes a focal spot with known dimensions, as well as an active area of the detector, into account. It also models the statistical distribution of the measured data from the physics of the interaction of X-rays with matter [5].

ASIR-V reconstruction is blended with traditional FBP in 10% increments until 100% as it can be initialized with a FBP to facilitate a fast convergence.

In recent years, DLIR has been developed as a subset of the development of artificial intelligence (AI) in the medical field. Algorithms based on deep learning actually exceed the limits of IR in terms of image noise reduction, further improving spatial resolution, and it is expected to reduce dose contribution, as demonstrated by previous studies with this focus. 

The DLIR technology used by the GE Healthcare is TrueFidelity™. It is based on a Deep Neural Network (DNN) that contains multiple layers of mathematical equations, which are trained to find the correct mathematical manipulation of images.

The starting point to train and verify a neural network is using a large set of high-quality projection data obtained by FBP algorithms at high doses (ideal model) from phantoms and clinical environments, which represent the ground truth.

In summary, a low-dose acquired sinogram is the input to the DNN, the output image is compared with the corresponding ground truth (FBP) in terms of several metrics, and the differences found are fed back into the engine. 

The training process is repeated on thousands of data points until the model becomes stable, including validation and testing supervised by image quality experts and experienced radiologists on a massive number of patient and phantom cases to cover different anatomies, scan conditions, and clinical indications [5].

During the algorithm commissioning process, we made a series of measurements as required by our quality control protocol to verify how the reconstruction with DLIR changes the main physical quantities, including the Hounsfield unit accuracy, contrast detectability, and noise and Noise Power Spectrum (NPS). 

DLIR has three reconstruction strength levels (low, medium, and high) to control the amount of algorithm intervention. Without impacting the reconstruction speed, the strength levels can be chosen in the protocols based on the clinical applications.

Using a high DLIR level can, in some cases, increase blurring. As an example, a study [6] showed how small lesion detectability on low-contrast organs makes it necessary to use a medium level of strength.

For the CCTA-specific clinical tasks, the radiologists chose to set a high level after a comparison of the outcome results on a Catphan^®^ 600 Phantom.

The raw CT data of all the patients of Group B were reconstructed by the ASIR-V at a level of 60% and the DLIR at a high level to obtain a set of images with a layer thickness of 0.625 mm. 

After reconstruction, all images were analyzed for image quality assessment and to rule out qualitatively inadequate ones. Group A images were observed using the Suitestensa PACS (Picture Archiving and Communication System) workstation, and Group B images were observed using the GE AW Server 3.2 workstation.

### 2.4. Radiation Dose Indices and Comparisons with DRLs

Dosimetric data collection was made through our dose tracking software: Radimetrics™ Enterprise Platform. We chose basic dose indices as relevant parameters, including CTDIvol (Volumetric Computed Tomography Dose Index), SSDE (Size-Specific Dose Estimation) and the total DLP for a 32 cm body phantom. The DLP equals the CT dose index, CTDIvol body, multiplied by the scan length for all patients.

We pulled out the DLPs both for the full examination, including localization and pre-monitoring sequences, and for the individual phases considered separately. 

The radiological exams of Group A did not provide a Radiation Dose Structured Report (RDSR), so the data were obtained through DICOM Header information associated with the images, which provided a less complete amount of data but was adequate for the purpose of this study.

The DLP_CCTA_ represents the radiation exposure due to CT angiography only. We reported SSDE only for CCTA sequence.

We finally compared the median value obtained with national and international DRLs, as known DRLs are not absolute limits or trigger levels and should not be used for individual patient optimization or a single examination [7]. 

A DRL is a level to aim for in a radiological examination; it does not replace professional judgment, and, above all, it is not to be used for self-referencing. 

It is rather a useful tool in a virtuous optimization process.

### 2.5. Effective Dose and Equivalent Dose to the Heart

The primary measure of radiation output for modern scanners is CTDIvol, an equipment-specific index based on the dose delivered to a polymethacrylate (PMMA) “phantom”, whose size is automatically selected at the time of the examination. The choice is based on the scanned anatomical region, but it does not represent the patient absorbed dose. However, CTDIvol is a good reference index because it is now mandatory in the console and in the RDSR, and it is reported for each single acquisition sequence.

The overall dose can be better estimated through Monte Carlo simulations, and it can be used to calculate the effective dose.

Effective dose concept was developed by the ICRP as a risk-adjusted dosimetric amount for the management of protection against stochastic effects.

Our dose tracking software allows us to evaluate, through a Monte Carlo-based dosimetry software, the effective dose and the equivalent dose to the heart.

In organ simulation, the whole phantom is scanned along the z-axis in a series of slices. At each slice position of CT scanning, the total estimated energy absorbed by each organ is recorded. Organ dose estimate is based on modeling the interactions of X-ray photons with matter and uses scanner-specific values; the scan length is based on the CTDIvol and DLP provided.

Scanner-specific values are based on the information provided by the equipment manufacturers, and the X-ray source spectrum for photon movement is based on the model described by the National Radiological Protection Board (NRPB) [8].

Phantoms used for human simulation are modeled through mathematical phantoms and the patient-to-phantom mapping is based on age and gender. 

The Radimetrics phantom library contains phantoms representing newborn to adult sizes. 

We do not consider effective dose to be a correct individual metric, but we do estimate and use it for optimization purposes when comparing different protocols.

Regarding the equivalent dose (H), we are aware that it is not required as a protective quantity but is more appropriate as a limit to avoid tissue reactions and should be set in terms of absorbed dose (Gy) rather than dose equivalent (Sv). We use it, like E, exclusively for optimization purposes. 

Effective and equivalent doses are calculated using a software according to the ICRP 103 [9] formulation and weighting factors. For all patients, these quantities are referred to in the entire examination and include the localizer and Smart Prep contributions.

In the patients of Group A, we were not able to estimate heart dose using a software as, after any kind of reconstruction, CT system did not provide scanned region identification, leaving the corresponding Digital Imaging and Communications in Medicine (DICOM) field empty. The absence of such input data made the calculation impossible.

As a further comparison, we also estimated the effective dose through DLP based on the effective dose conversion factor found in the literature for our specific type of exam [10] and evaluated the difference between this standard method and the Monte Carlo-simulated calculations from the software engine.

## 3. Results

According to the inclusion criteria mentioned in the previous section, 40 patients (20 for each group) were finally selected.

Group A has a mean age of 67.7 (42–85) years, with anatomical values of 75.1 (58–112) kg as the mean weight and 168 (148–189) cm as the mean height. 

Group B has a mean age of 59.9 (28–81) years, with anatomical values of 78.9 (53–103) kg as the mean weight and 165.3 (150–185) cm as the mean height. 

In Table 2, we summarize the results of the calculated median values for DLPs DLP_CCTA_, and SSDE. The median value is the statistical index recommended by the ICRP for comparisons with DRLs.

The mean effective dose of the whole protocol as calculated from the Monte Carlo simulation is 32.8 mSv for Group A and 5.6 mSv for Group B. 

Regarding the effective dose estimates, we are very confident in the results provided by the Monte Carlo simulation software. As part of our quality control program of the dose tracking system, we periodically verify that the automatic choice of Monte Carlo phantoms is in accordance with sex, age, and effective diameter of patients; the last is obtained from localizer images. In the case of CCTA exam, scout images are acquired in correspondence with the scanned thoracic portion, making the estimate of the effective diameter more reliable.

We then estimated the effective dose based on cardiac DLP with the effective dose conversion factor of 0.026 mSv·mGy^−1^·cm^−1^ [10]; for Group A, the observed DLP of 1158 mGy·cm corresponds to an effective dose of 29.9 mSv, and for Group B, the observed DLP of 221 mGy·cm corresponds to an effective dose of 6.0 mSv.

The difference between the standard formula and the Monte Carlo simulation is not statistically significant (*p* < 0.01); the standard deviation of the difference between the two methods is 0.3 mSv.

The mean equivalent dose to heart of the whole protocol as calculated from the Monte Carlo simulation is 97.3 mSv for Group A and 20.5 mSv for Group B.

In Table 3, we report the reference DRLs from the literature [11,12,13]. The Italian DRL represents the reference value to be taken into account according to the Italian legislation on radiation protection, but the value comes from studies with tests that were not performed with optimized techniques [11]. Then, we considered the United Kingdom’s DRL for CCTA, which comes from a recently performed national dose survey [12] taking into account data from fifty centers with a total of 1341 coronary CTA exams. In the survey, the median acquisition heart rate and exam DLP were 60 bpm and 209 mGy·cm, respectively, and the corresponding effective dose was estimated as 5.9 mSv using a conversion factor of 0.028 mSv^−1^·mGy·cm, which was slightly different from the one chosen in our study.

The UK National Diagnostic Reference Levels (NDRLs) had been updated on 24 November 2022.

Radiologist assessment of the post-processing images coming from the two types of reconstructions was performed on a small subset of Group B patients using the AW server 3.2 workstation (GE Healthcare).

Vessel morphology and plaque characteristics were compared, and the results affirm that DLIR-reconstructed images show decreased image noise and improved image quality with a better definition of anatomical borders and edges, providing detailed findings in a study of normal and pathological coronary walls.

**Figure 1:**
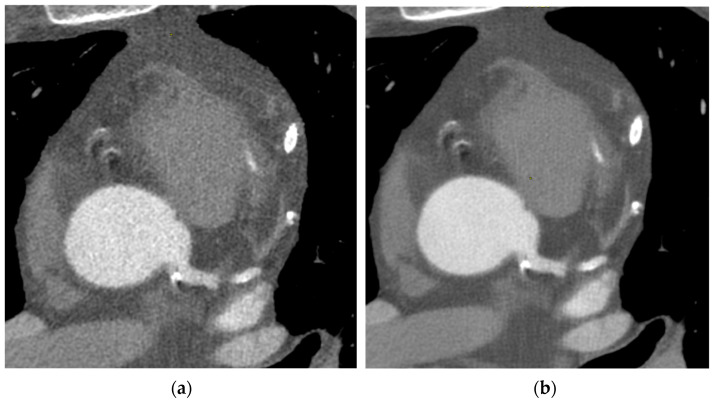
Clinical images of a patient’s CCTA sequence reconstructed with the ASIR-V algorithm at 60% (**a**) and DLIR algorithm at high level (**b**).

As we can see in Figure 1, the DLIR (b) shows image quality improvement with reduced image noise and better-defined anatomical details, when compared with the ASIR-V(a).

In fact, the contrast enhancement of the aorta and the left coronary artery is more homogeneous with the disappearance of multiple small areas of hypodensity and a consequent better visualization of the vessels’ boundaries and internal structure, which are the fundamental details in the study of the coronary wall.

Radiologists have found that coronary arteries have better visualization in both calcified and soft plaques, which leads to a higher level of confidence when assessing stenosis.

The images also show an improvement in the measurement of the bifurcation angle on the three-dimensional volume-rendered image of the coronary artery tree.

As an additional preliminary result, an evaluation of the image quality allows us to state that the DLIR algorithm reconstruction gives better results than that of the ASIR-V alone. 

It combines the measurement using the Catphan^®^ 600 Phantom as part of the quality checks carried out for scanner acceptance and the opinion of radiologists.

The Catphan^®^ 600 analysis mainly showed a noise reduction, an improvement in CNR (contrast-to–noise ratio), and low-contrast detectability. 

As reported in an algorithm technical paper [5], we found that the effect of the algorithm lies in its ability to modify the noise spectrum, assimilating the noise spectrum to the typical one of the FBP.

The NNPS analysis was performed using the imQuest CT image analysis tool developed by Duke University. 

## 4. Discussion

The CCTA is a growing exam as it provides a solution for many clinical questions and shows better cost-effective benefits than conventional coronary angiography in the study of suspected coronary artery disease.

The preliminary study of our actual technique and the dosimetric data have shown remarkable results in terms of patient dose reduction. 

A statistical comparison between the results of the two protocols is meaningless given the difference between the technologies, mainly due to the total absence of an iterative algorithm in the old scanner.

However, the decrease in voltage compared to the previous technology is promising to us because a multicenter study [2] underlined that a reduction in tube potential is extremely effective as it combines dose reduction with an increase in iodine absorption, giving rise to the advantages in iodinated contrast-enhanced CCTA imaging.

More recent studies [14,15] lead to testing protocols at very low voltages (70 kV), which would also allow optimizing the amount of injected contrast medium.

Additionally, the first feedback of the radiologists in the scanner transition is that TrueFidelity™-reconstructed images of vessels and plaque in CCTA show improved image quality if compared to previous algorithm reconstructions used.

The preliminary measurements of physical parameters using the Catphan^®^ 600 phantom within our quality assurance program also support the qualitative impression, combining an improvement in physical index values with ionizing radiation dose reduction.

Referring to the literature, the efficacy of DLIR for dose reduction in CCTA has also been demonstrated in a previous study [16], which highlighted how the intervention of the deep learning algorithm reduces the dose without altering noise and improves the contrast-to-noise ratio (CNR). This study also showed how the estimates of the severity of stenosis and the composition and volume of the plaques are corrected despite the intervention using the DLIR algorithm. 

The explicit decrease in the DLP values and their comparison with the European DRLs encourage us to go on with the optimization work.

A study to further improve the CCTA protocol is planned in our hospital; we will perform a task-based image quality assessment and detectability comparisons in accordance with the American Association of Physicists in Medicine (AAPM) TG 233 report [17]. The method was introduced by the AAPM to overcome the impossibility of correctly evaluate modern algorithms with traditional metrics (i.e., noise as standard deviation or signal-to-noise-ratio). We will compare the results based on Noise Index (NI) variation.

For GE scanners, NI is the primary image quality parameter; according to its choice, the tube current used for the acquisition window is automatically delivered by the system. 

As can be guessed, for the same NI, increased ASIR-V levels tend to reduce tube output. The main difficulty in optimizing imaging through NI for our specific clinical task lies in the fact that, in the GE Revolution scanner series, the parameter incorporates for the first time the intervention of ASIR-V in the primary reconstruction. 

For this reason, it will be challenging to consider the contribution of each individual algorithm [18]. As a result of this study, we found that CT automatisms were adjusted with a median noise index of 13.9 for Group B, without the intervention of the operator who carried out the examination.

Further radiation dose reduction will be possible with the implementation of an auto-gated EGC protocol. Through this protocol, the system automatically adjusts the scan parameters based on patients’ heart rate and heart rate variability during the scan, and it acquires the scan at the highest mA load in a very narrow window of the cardiac cycle.

## 5. Conclusions

The CCTA is a growing exam as it provides a solution for many clinical questions. In this work, we compared our current scanning technique with the old one.

A major technological upgrade allowed us to improve image quality and reduce patient dose.

We analyzed the dosimetric data of 2 groups of 20 patients, and the results showed a large reduction in radiation dose.

The importance of these results lie in the fact that a decrease in radiation dose means a reduction in patient detriment, especially with increasingly widespread examinations such as the CCTA.

Our future aim is to improve the quality of CT exam through physical optimization of the exposure parameters using detectability index metrics. We will also tune the intervention levels of the reconstruction algorithms, as they highly impact the final results. Another step will be the use of an auto-gated protocol to acquire a scan at a higher mA load in a narrower window of the cardiac cycle.

## Figures and Tables

**Table 1:** Acquisition parameters in the two patient groups.

Scan Parameter	Group A	Group B
Scan technique	Helical	Axial
Pitch factor	0.18	1
Rotation time (s)	1.7	0.28
kV	140	100/120 **
mAs *	813	155
Scan length_CCTA_ *** (mm)	182 (171–190)	160

* Mean values. ** Variable between the two values according to the patient effective diameter. *** Scan lenght_CCTA_ refers to the CT angiography sequence only.

**Table 2:** Median values for the dosimetric parameters in the two patient groups.

Parameter	Group A	Group B
CTDIvol (mGy)	62.6	12.8 (4.6–30.1)
SSDE (mGy·cm)	87.7	17.1 (7.7–38.2)
Total DLP (mGy·cm)	1158 (1024–1336)	221 (82–488)
DLP_CCTA_ (mGy·cm)	1140 (1072–1308)	204 (74-481)
Effective dose from MC simulation (mSv)	32.8	5.6
Effective dose from conversion factor (mSv)	29.9	6.0
Equivalent dose to heart (mSv)	- *	20.5

* Heart equivalent dose is not computable by the software as the header DICOM does not provide the scanned target region.

**Table 3:** Reference DLPs for CCTA (mGy·cm).

Country	Dose-Length Product (DRL) for CCTA (mGy·cm)
Italy	1208
France	870
UK	209

## Data Availability

The data presented in this study are available from the corresponding author upon request.
